# Signet-ring cell carcinoma in rectal malignancies: a case report with an unexpected outcome

**DOI:** 10.3389/fonc.2025.1516220

**Published:** 2025-05-26

**Authors:** Aichun Long, Cheng Huang, Qinwei Xu, Shasha He, Li Zhang, Cuiwei Zhang

**Affiliations:** ^1^ Department of Pathology, The Affiliated Hospital of Southwest Medical University, Luzhou, Sichuan, China; ^2^ School of Basic Medical Sciences, Southwest Medical University, Luzhou, Sichuan, China; ^3^ Endoscopy Center, Department of Gastroenterology, Shanghai East Hospital, School of Medicine, Tongji University, Shanghai, China; ^4^ Department of Radiology, The Affiliated Hospital of Southwest Medical University, Luzhou, Sichuan, China; ^5^ Department of Pathology, Shanghai East Hospital, School of Medicine, Tongji University, Shanghai, China

**Keywords:** signet-ring cells, genetic testing, prostate cancer, SPOP, BRAF

## Abstract

Signet ring-like cell prostate cancer is a rare and highly aggressive histological subtype of prostate cancer. The characteristic feature is that the cancer cells produce a large amount of mucin. This mucin gradually accumulates within the cells, pushing the nucleus to one side and giving the cells a signet-ring-like appearance. Late-stage prostate cancer typically metastasizes to bones, lungs, and other organs via lymphatic or hematogenous routes. Rectal involvement with gastrointestinal symptoms as the initial presenting manifestation is relatively uncommon. This case report aims to describe the diagnostic process of prostate cancer in an 82-year-old male patient. The initial symptom was diarrhea, the admission diagnosis was rectal malignancy, and the diagnostic clue was the presence of signet-ring cells. The tumor origin analysis was conducted alongside serological and imaging examinations. Furthermore, this report discusses the endoscopic manifestations, pathomorphological changes, and clinical prognostic analysis of this prostate cancer case, to enhance the recognition of rare pathological subtypes of prostate cancer.

## Introduction

Prostate cancer is a common malignancy in the male population (1–3)and a leading cause of cancer-related deaths in men (4–8). Its incidence is increasing annually (9, 10). Prostate cancer is most commonly diagnosed in men over the age of 50. It has a relatively favorable 5-year survival rate, mainly due to early detection and the availability of radical surgical treatments (11). However, the majority of prostate cancer-related mortalities are attributed to the progression of the disease to a metastatic state and the subsequent emergence of treatment resistance. Although prostate cancer is most commonly presented as adenocarcinoma, its rare pathological variants exhibit unique pathological characteristics and prognostic outcomes. Signet-ring cell prostate cancer is a rare, poorly differentiated, and aggressive histological variant characterized by the presence of signet-ring cells (12–14). The diagnosis of signet-ring cell adenocarcinoma can be made when signet-ring tumor cells comprise at least 25% (15–18).

Signet ring-like cell prostate cancer is often associated with an irregular tumor growth pattern and a pronounced propensity for metastasis. Studies suggest that this type of cancer may exhibit biological behavior distinct from that of common prostate cancer, showing higher metastasis rates and poorer prognosis in advanced stages. The pure Signet ring-like cell prostate cancer subtype is extremely rare and often mixed with other types. Intracytoplasmic vacuoles in cells are not exclusive to any specific Gleason grade. However, they are more frequently observed in cases with higher Gleason grades, often correlating with a worse prognosis. Germline pathogenic variants have been identified in up to 15% of prostate cancer patients, particularly in those with metastatic disease. Genetic testing may help identify potential options for precision therapy and reveal a range of hereditary cancer susceptibility syndromes with distinct clinical features (19). This report presents a rare case of Signet ring-like cell prostate cancer with rectal involvement. The endoscopic manifestations and pathomorphologic changes observed in this case are documented, along with the mutated genes identified through genetic testing and the clinical prognostic analyses conducted to enhance understanding of rare pathological subtypes of prostate cancer. This report not only enriches the knowledge base for the recognizing prostate cancer and its rare subtypes but also provides valuable insights in the diagnostic process of clinical pathology.

## Case report

### The clinical data

**Figure 1:**
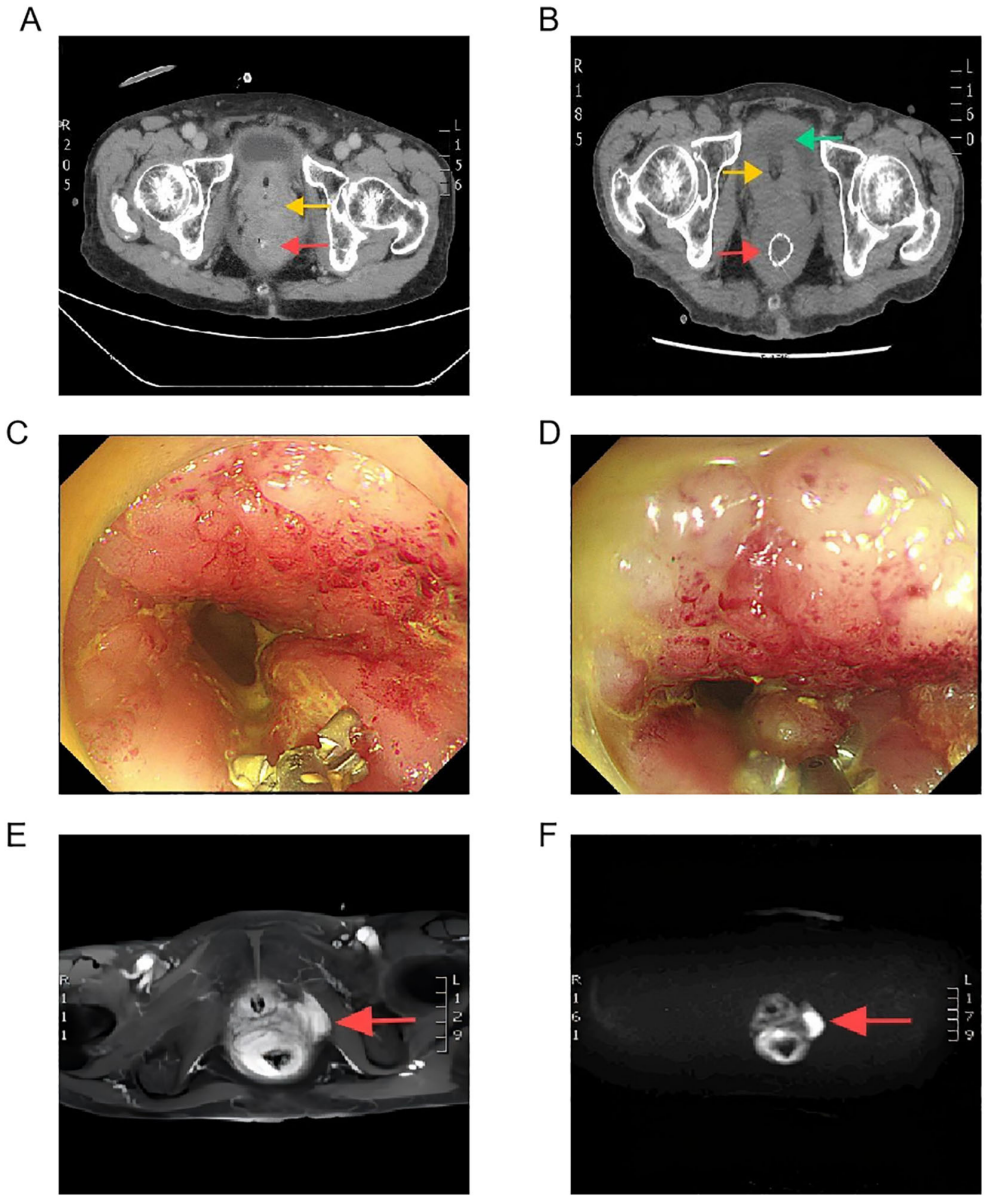
Results of imaging studies and endoscopic examination. **(A)** Contrast-enhanced CT of the lower abdomen.The red arrow indicates: thickened rectal wall; the yellow arrow indicates: prostate-rectal junction. **(B)** Non-contrast 64-slice CT of the lower abdomen. The red arrow indicates a thickened rectal wall; the yellow arrow indicates the prostate; the green arrow indicates the bladder. **(C, D)** Electron colonoscopy revealed rectal edema and luminal obstruction. **(E)** T1-weighted imaging (T1WI) sequence with contrast enhancement. The encircled region represents the lesion at its maximum dimension. **(F)** Diffusion-weighted imaging (DWI) sequence. The encircled region represents the lesion at its maximum dimension.

An 82-year-old male patient presented to the emergency department of our hospital on May 14, 2023, with a primary complaint of diarrhea lasting more than 10 days accompanied by fatigue. A diagnosis of gastrointestinal dysfunction was made, and treatment was initiated with Cefoperazone sodium, Levofloxacin sodium chloride for infection, and Esomeprazole sodium for acid suppression and gastric protection, along with other symptomatic therapies. Despite these interventions, the patient reported no improvement in symptoms. Subsequently, the patient visited Jinyang Community Health Hospital, where a fecal occult blood test returned negative. For further evaluation and treatment, the patient was referred to our hospital and admitted with an outpatient diagnosis of diarrhea. On May 20, 2023, serological examination revealed reduced levels of erythrocytes (2.91×10^12/L), albumin (34 g/L), cholinesterase (2781 U/L), and potassium (1.7 mmol/L). Additionally, urinalysis showed elevated microscopic erythrocytes, urinary occult blood (4+), and a positive fecal occult blood test (using the colloidal gold method). Laboratory and imaging studies upon admission are summarized in **Supplementary Table 1**.On May 23, 2023, the patient tested positive for the COVID-19 nucleic acid. Given the positive result and the clinical presentation, it was considered that the diarrhea might have been induced by COVID-19. The patient exhibited positive fecal occult blood, attributed to local mucosal shedding in the intestines, likely caused by frequent diarrhea (more than 10 episodes daily) over the past two weeks. The patient was prescribed oral Nirmatrelvir for antiviral treatment and Montelukast for symptomatic antidiarrheal management, along with other supportive therapies. On May 24, 2023, a routine urological examination revealed potential benign prostatic hyperplasia with calcification, a left renal cyst, mild left hydronephrosis, dilatation of the upper left ureter, mild separation of the right renal pelvis, and possible urinary retention. Subsequently, the patient developed urinary retention and bilateral lower limb edema. An indwelling urinary catheter was inserted, and diuretics, along with other symptomatic treatments, were administered. The patient has a history of gout and is routinely prescribed oral Febuxostat or Benzbromarone (1 tablet daily). Thirty years ago, the patient underwent an appendectomy, although the details of the procedure remain unclear. The patient denies any family history of hereditary diseases. On May 25, 2023, enhanced abdominal CT imaging revealed asymmetrical thickening of the lower rectal wall, along with enlarged lymph nodes around the rectum and adjacent to the right iliac vessels. The borders of the prostate and left seminal vesicle were indistinct, with benign prostatic hyperplasia and calcification noted (**Figure 1A**). On June 1, 2023, an electronic colonoscopy identified a 0.8 cm subpedunculated polyp in the transverse colon, which was subsequently excised via endoscopic mucosal resection (EMR). Additionally, an ulcer was observed in the sigmoid colon. Symptomatic treatment was provided, and the patient’s condition improved, with no abdominal pain, distention, hematemesis, or melena. The patient was deemed fit for discharge. On August 29, 2023, the patient presented with complaints of difficulty defecating, abdominal distension, and exacerbated abdominal pain over the past two days. For further diagnosis and treatment, the patient was re-admitted with clinical suspicion of intestinal obstruction. On physical examination, the abdomen was soft, with a visible, healed surgical scar, but no palpable masses or bowel sounds. There was no tenderness, rebound tenderness, or muscle rigidity. McBurney’s point was non-tender, and bowel sounds were not hyperactive. No shifting dullness was observed. Examination of the anus, rectum, and external genitalia was not performed. A follow-up lower abdominal CT scan (64-slice, non-contrast) in September 2023 revealed rectal stent placement, with the remaining diagnostic findings consistent with the enhanced CT from May (**Figure 1B**). On September 7, 2023, colonoscopy revealed rectal edema, pinpoint stenosis, obstruction of the intestinal lumen, and proximal intestinal extension. These findings were observed despite the colonoscope barely passing through the colon (**Figures 1C, D**). Consequently, a colonoscopic diagnosis of perirectal circumferential stenosis with obstruction was made, followed by endoscopic stenting and biopsy.

**Figure 2:**
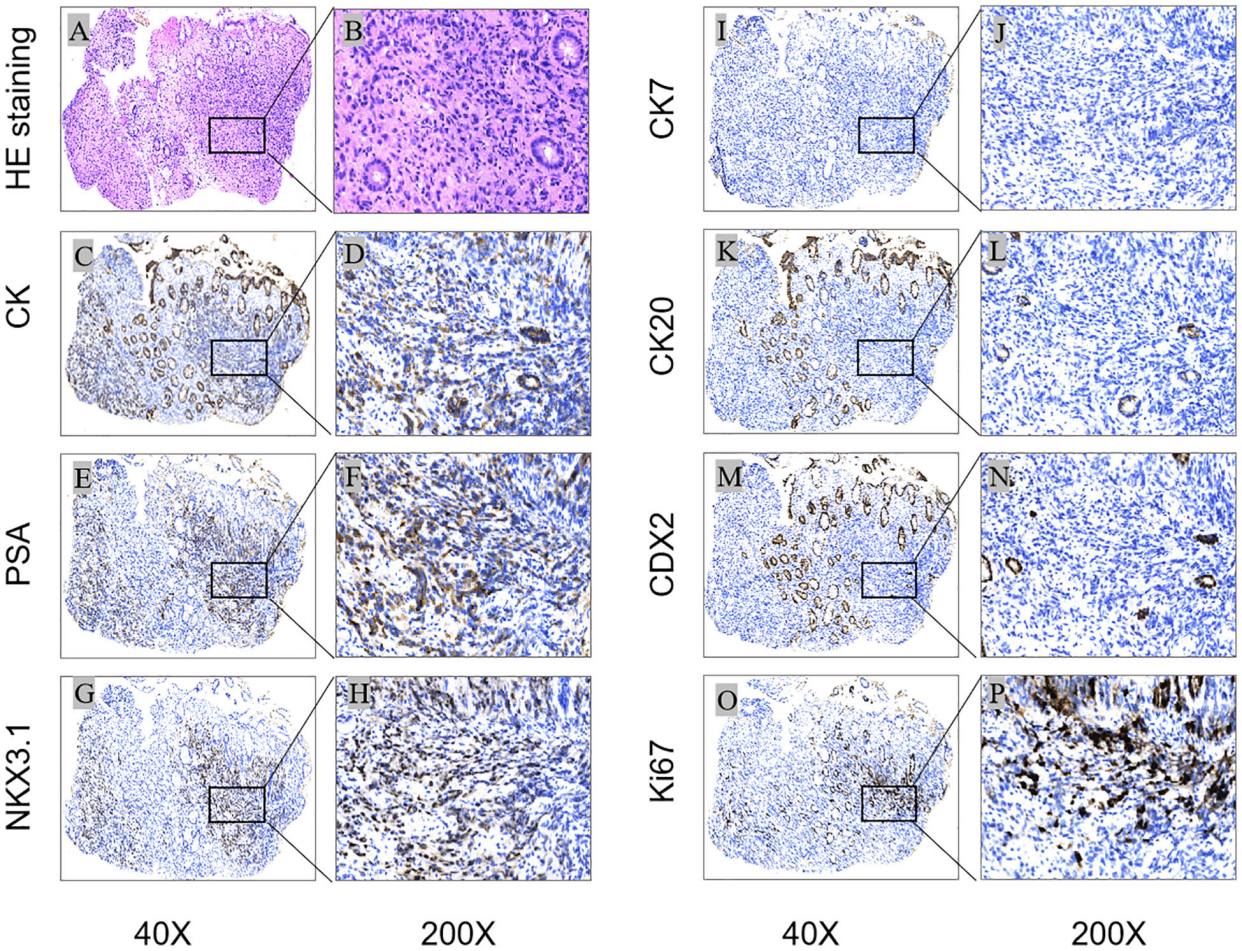
Histological and immunohistochemical results: Microscopic finding in rectal biopsy showing an infiltration of the intrinsic layer by signet ring cells [**(A)**:HEx40] [**(B)**HEx200]; The cells were positive for CK, PSA and NKX3.1 [**(C, E, G)**x40] [**(D, F, H)**x200], Ki-67 proliferation index was approximately 10%[**(O)**x40] (Px200); staining for CK7, CK20 and CDX2 was negative [**(I, K, M)**x40] [**(J, L, N)**x200]. [**(A, B)** (HEstaining); **(C–P)** (IHC); **(C, D)** (CK), **(E, F)** (PSA), **(G, H)** (NKX3.1), **(I, J)** (CK7), **(K, L)** (CK20), **(M, N)** (CDX2), **(O, P)** (Ki-67)].

Histopathology revealed that the intrinsic glandular epithelium did not exhibit evidence of heterogeneous hyperplasia or migration in conjunction with the tumor cells. The tumor cells exhibited invasive growth and were characterized by signet-ring cell morphology. The initial pathological diagnosis was poorly differentiated adenocarcinoma. Since signet-ring cells are not exclusive to gastric and intestinal adenocarcinomas, and imaging revealed that the prostate gland lacked clear demarcation from the surrounding structures, immunohistochemistry was performed. The immunohistochemical analysis results were as follows: CK (+), PSA (+), NKX3.1 (+), CK7 (–), CK20 (–), CDX-2 (–), GATA3 (–), and Ki-67 (+, approximately 10%) (**Figure 2**). The final pathological diagnosis was prostate cancer with rectal involvement, a Gleason score of 5 + 5 = 10, and ISUP grouping 5. Based on the pathological diagnostic results, the patient underwent further testing for tumor markers, namely PSA (282.00 ng/ml), NSE (17.4 ng/ml), and CA24-2 (12 IU/ml).

### MRI enhancement

The results of the imaging examination were consistent with the expected findings. The prostate gland exhibited abnormal signal intensity with poorly defined borders, presenting as low T1WI, mixed T2WI, and a high signal shadow in DWI. Additionally, the gland displayed markedly inhomogeneous enhancement after contrast administration. (**Figures 1E, F**) revealed high signal shadows in DWI on both sides of the seminal vesicle glands, irregular thickening of the rectal wall, and a swollen, enlarged lymph node-bearing pelvic region. Radiologic diagnosis: Prostate lesion, prostate cancer highly suspected, bilateral involvement of the seminal vesicle glands, rectal involvement, and pelvic lymph node metastasis.

### DNA sequencing and data analysis

To explore the key molecular events leading to the malignant biological behavior of prostate cancer, second-generation DNA sequencing on tissue samples was performed at the Nanjing Shihe Medical Testing Laboratory. The aim was to identify rare variants associated with the metastasis and progression of Signet ring-like cell prostate cancer. The results identified two significant mutations linked to tumorigenesis in the tested samples: the V600E mutation in *BRAF* and the F133L mutation in *SPOP*. The *BRAF* mutation is located in exon 15. This missense mutation is characterized by a change from T to A at nucleotide position 1799, resulting in an amino acid change at position 600 from valine to glutamate (V600E). The *SPOP* gene mutation, located in exon 5, is characterized by a change from T to C at nucleotide position 397, resulting in the alteration of phenylalanine to leucine (F133L) at the amino acid level.

**Figure 3:**
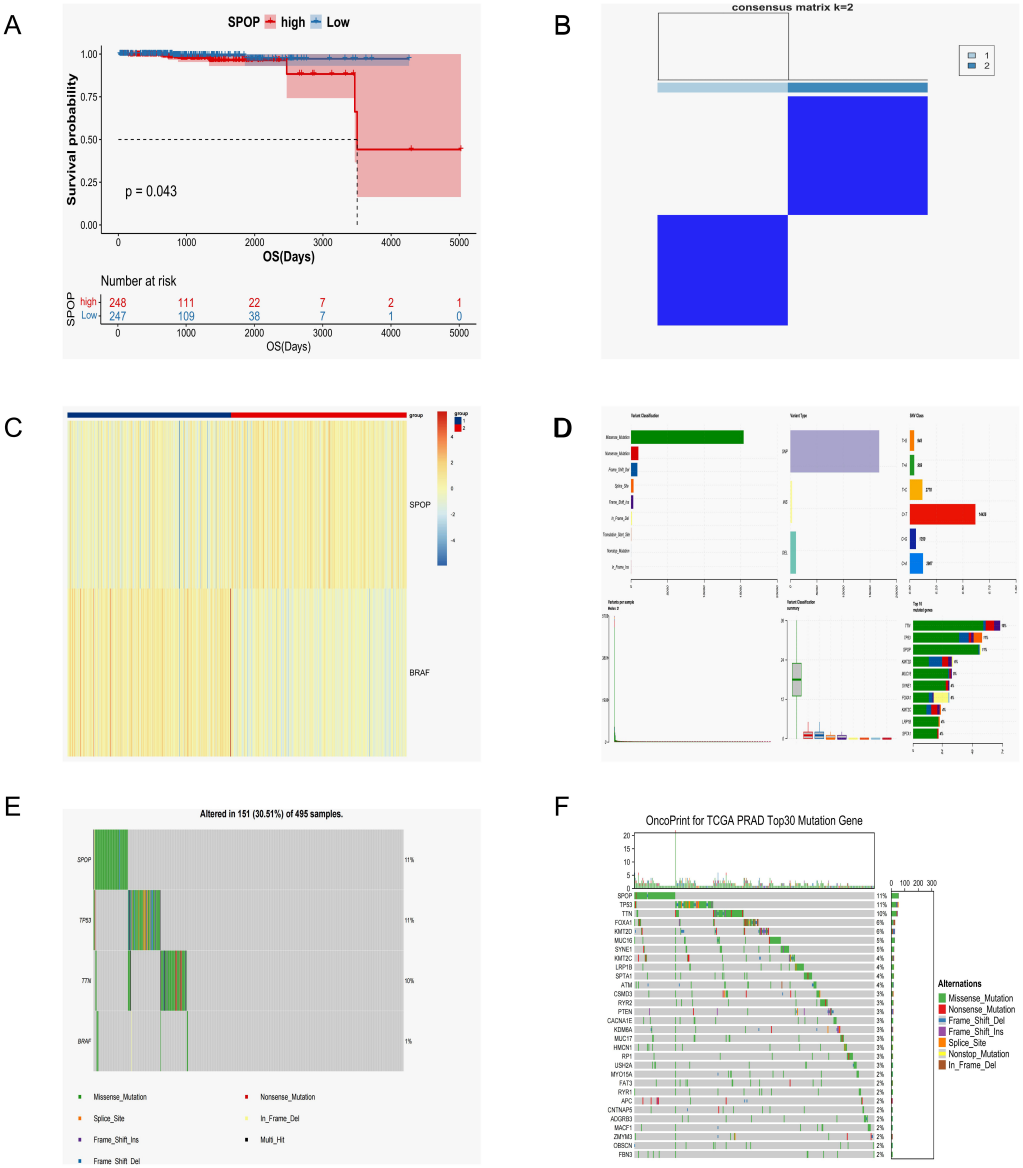
The sample analysis results from the Sangerbox data platform. **(A)** *SPOP* Survival Curve Analysis. **(B)** Consistency clustering grouping information. **(C)** Consistency clustering heat map analysis. **(D)** Top 10 mutated genes and their mutation types in prostate cancer samples. **(E)** *SPOP* and *BRAF* mutant gene types and their percentages. **(F)** Waterfall map of the top 30 mutations in prostate cancer samples.

To clarify the impact of *BRAF* and *SPOP* mutations on prostate cancer patients’ survival time, the Sangerbox data platform (http://sangerbox.com/home.html) was used to download the mRNA expression matrix and clinical survival data of prostate cancer samples. After excluding cases with missing clinical information, 495 samples were retained for further analysis. The *SPOP* expression, divided by the median, was used to categorize the samples into high and low -expression groups. Subsequently, Kaplan-Meier survival curves were generated using the R survival package (**Figure 3A**). The results showed that the survival rate was diminished in the *SPOP* high-expression cohort, with a statistically significant difference between the two groups (p=0.043).

Transcriptome data were then downloaded from the TCGA database, and the expression profiles of prostate cancer tumor samples (n=483) were divided into two groups (group 1 and group 2, consensus matrix k=2) based on *SPOP* and *BRAF* gene expression using the consistency clustering algorithm (**Figure 3B**). The ConsensusClusterPlus software package was used to generate a consensus clustering heatmap analysis (**Figure 3C**), showing the degree of similarity between samples. The results showed notable differences between the two groups, with a high degree of consistency.

Finally, samples of masked somatic mutations (MSM) in single nucleotide variation (SNV, n=501) were downloaded from the TCGA database for further analysis alongside the sequencing results. The results show that in prostate cancer, mutations are more likely to occur in *SPOP*, TP53, and TTN, with the *SPOP* gene being more prone to missense mutations than other mutation types (**Figures 3D-F**). Unfortunately, the patient has not undergone surgical treatment at our institution due to personal reasons. We will continue to monitor the patient through regular follow-up visits and have advised the patient to routinely monitor serum PSA, NSA, creatinine, hemoglobin, liver function, and testosterone levels, as well as undergo CTC, DRE, sexual function/urinary control evaluations every 2–6 months. Additionally, abdominal and pelvic CT or MRI should be performed at least annually.

## Discussion

Signet ring cell carcinoma is characterized by high invasiveness and poor prognosis. Although it can occur in various organs, including the stomach, colon, esophagus, bladder, prostate, pancreas, and breast (20), it is most commonly found in the gastrointestinal tract (13). Genetic testing in this case, mutations in the *SPOP* and *BRAF* genes were identified. These mutations are linked to an increased risk of tumor formation, proliferation, metastasis, and a diminished response to therapy. Our analysis of database samples showed that mutations in the *SPOP* gene are associated with a poor prognosis. This suggests that the expression level of *SPOP* significantly influences the survival outcome of patients. Gene sequencing provides essential biomarker information, suggesting that these mutations may be linked to the tumor metastasis process. The *BRAF* gene mutation identified in this case is a missense mutation at codon p.V600E in exon 15, within the kinase domain of the *BRAF* protein. This mutation is a common hotspot in *BRAF* and has been reported in various cancers, including melanoma, lung cancer, and colorectal cancer. It results in the constitutive activation of *BRAF* kinase activity, which subsequently promotes downstream MEK/ERK signaling, contributing to tumorigenesis. *BRAF* Class I mutations may benefit from combination treatment with RAF monomer inhibitors and downstream MEK/ERK inhibitors. This helps reveal potential driver mutations and candidate therapeutic targets, offering new insights into predicting prostate cancer immunotherapy in the future.

The advent of high-throughput gene sequencing technology has enabled comprehensive sequencing of numerous DNA and RNA samples in a relatively short timeframe. This technological advancement is expected to propel genomics research forward, enhancing our understanding of underlying genetic mechanisms. The MAPK/ERK signaling pathway plays a crucial role in regulating various cellular processes, including cell growth, proliferation, differentiation, and apoptosis. The proto-oncogene *BRAF* has been identified in a wide range of tumors, with oncogenic *BRAF* mutations found in approximately 6% of human cancers. Oncogenic *BRAF* mutations lead to constitutive activation of the MAPK/ERK pathway, resulting in uncontrolled proliferation, survival, invasiveness, and drug resistance of tumor cells (21, 22). *BRAF* alterations have been reported in 3% to 5% of prostate cancers. The majority of these alterations are class II mutations and rearrangements, with *BRAF* V600E mutations being extremely rare (23). A number of studies have supported the assertion that *SPOP* serves as a substrate adaptor for cullin 3-based E3 ligases, thereby playing a pivotal role in various cellular processes by targeting proteins for ubiquitination and subsequent proteasomal degradation (24). *BRAF* serves as a substrate for *SPOP*, which facilitates the non-degradative ubiquitination of *BRAF*, leading to the inactivation of the MAPK/ERK pathway. However, it have been shown that prostate cancer-associated *SPOP* mutations result in aberrant activation of the MAPK/ERK pathway in a *BRAF*-dependent manner, potentially contributing to the malignant transformation of cancer cells (22).

The risk of developing prostate cancer is associated with several factors, including age, race, family history, prostate-specific antigen (PSA) levels, the ratio of free to total PSA, and rectal findings (25). A comprehensive evaluation of clinical, gastrointestinal endoscopic, imaging, and histopathologic aspects was required, necessitating a multidisciplinary diagnostic approach. Imaging techniques, including enhanced CT and MRI, enable visualization of a thickened rectal wall and enlarged lymph nodes, which may suggest malignancy. However, imaging does not specify the site of origin. The diagnosis of metastatic prostate cancer was confirmed through endoscopic biopsy and immunohistochemical staining, with positivity for PSA and NKX3.1. These markers confirmed involvement in prostate cancer, distinguishing it from signet ring cell carcinoma originating in the gastrointestinal tract. Consequently, this case exemplifies the pivotal role of immunohistochemistry in determining the primary site of cancerous growth.

Due to its aggressive nature and resistance to treatment, researching the mechanisms of prostate cancer metastasis remains challenging. Traditional treatments for prostate cancer, including androgen deprivation therapy (26, 27) and chemotherapy (28), may have limited efficacy in this context. The identification of mutations in the *BRAF* and *SPOP* genes through genomic sequencing offers the potential for predicting the response to prostate cancer immunotherapy, assessing prognosis, and targeting therapy. Inhibitors targeting the MAPK/ERK pathway, such as *BRAF* inhibitors, represent a promising avenue for therapeutic intervention in patients with these mutations (29). This report provides a detailed diagnostic process and molecular insights into a rare case of signet ring-like cell prostate cancer with rectal involvement, emphasizing the importance of comprehensive diagnostic evaluation and the potential for targeted molecular therapies. Through a multidisciplinary diagnostic approach, including endoscopic examination, imaging studies, histopathology, and immunohistochemistry, an accurate diagnosis of this aggressive prostate cancer subtype was established.

## Conclusion

In conclusion, this case highlights the importance of continued research and multidisciplinary collaboration in advancing the understanding and management of rare and aggressive malignant tumors, with the ultimate aim of improving patient outcomes.

## Data Availability

The raw data supporting the conclusions of this article will be made available by the authors, without undue reservation.
