# Supplementary material for: Signet-ring cell carcinoma in rectal malignancies: a case report with an unexpected outcome
Source: Front Oncol. 2025 May 26;15:1516220. doi: 10.3389/fonc.2025.1516220 (PMC12146205; doi:10.3389/fonc.2025.1516220)
Supplement: Supplementary file 1 [file Table1.docx]

Supplementary Table 1 Clinical Timeline

| Date | Procedure | Findings |
| --- | --- | --- |
| 2023.5.20 | Urinalysis;faecal routine | Urine Occult Blood 4+ ↑, Leukocytes 6-9/HP, Occult Blood Test (Colloidal Gold) Positive ↑. |
| 2023.05.23 | Epstein-Barr virus (EBV) 6 | EBV VCA antibody IgG 50.00 Positive AU/mL ,EBV nuclear antigen IgG antibody 2.71 Positive AU/mL. |
| 2023.05.24 | Male Urinary Routine | Prostatic hyperplasia with possible calcification and dilatation of the upper left ureter. |
| 2023.05.25 | Lower abdomen (CT-enhanced 64-slice) | Asymmetric thickening of the wall of the lower rectum, enlargement of the perirectal and right iliac paravascular lymph nodes; unclear demarcation of the prostate and left seminal vesicle glands. |
| 2023.08.29 | Electron colonoscopy;biopsy. | Circumferential rectal stenosis with obstruction, endoscopic tube placement. |
| 2023.09.12 | HE+IHC | HE:poorly-differentiated carcinoma  IHC:Consistent with metastatic/invasive prostate cancer, Gleason score 5+5=10, ISUP subgroup 5. IHC results: CK(+), PSA (+), NKX3.1 (+), Ki-67 (+,10%). |
| 2023.09.14 | MRI enhanced | Prostate gland occupying, consider prostate cancer may be big, both sides of the seminal vesicle glands, rectal involvement, pelvic lymph node metastases. |
| 2023.09.14 | whole-body bone image | No obvious metastatic lesions on bones throughout the body. |
